# Fetal inflammation induces acute immune tolerance in the neonatal rat hippocampus

**DOI:** 10.1186/s12974-021-02119-w

**Published:** 2021-03-11

**Authors:** Garima Singh, Bradley J. Segura, Michael K. Georgieff, Tate Gisslen

**Affiliations:** 1 Division of Neonatology, Department of Pediatrics, University of Minnesota, East Building MB630, 2450 Riverside Avenue, Minneapolis, MN 55454 USA; 2 Division of Pediatric Surgery, Department of Surgery, University of Minnesota, East Building MB630, 2450 Riverside Avenue, Minneapolis, MN 55454 USA

**Keywords:** Preterm, Hippocampus, Microglial activation, Immune tolerance, LPS, Fetal inflammation

## Abstract

**Background:**

Infants born preterm due to chorioamnionitis are frequently affected by a fetal inflammatory response syndrome (FIRS) and then by subsequent postnatal infections. FIRS and postnatal systemic inflammatory events independently contribute to poor neurocognitive outcomes of preterm infants. Developmental integrity of the hippocampus is crucial for intact neurocognitive outcomes in preterms and hippocampally dependent behaviors are particularly vulnerable to preterm systemic inflammation. How FIRS modulates the hippocampal immune response to acute postnatal inflammatory events is not well understood.

**Methods:**

Prenatal LPS exposed (FIRS) and control neonatal rats received i.p. LPS or saline at postnatal day (P) 5. On P7, immune response was evaluated in the hippocampus of four treatment groups by measuring gene expression of inflammatory mediators and cytosolic and nuclear NFκB pathway proteins. Microglial activation was determined by CD11b+ and Iba1+ immunohistochemistry (IHC) and inflammatory gene expression of isolated microglia. Astrocyte reactivity was measured using Gfap+ IHC.

**Results:**

Postnatal LPS resulted in a robust hippocampal inflammatory response. In contrast, FIRS induced by prenatal LPS attenuated the response to postnatal LPS exposure, evidenced by decreased gene expression of inflammatory mediators, decreased nuclear NFκB p65 protein, and fewer activated CD11b+ and Iba1+ microglia. Isolated microglia demonstrated inflammatory gene upregulation to postnatal LPS without evidence of immune tolerance by prenatal LPS.

**Conclusion:**

Prenatal LPS exposure induced immune tolerance to subsequent postnatal LPS exposure in the hippocampus. Microglia demonstrate a robust inflammatory response to postnatal LPS, but only a partial immune tolerance response.

**Supplementary Information:**

The online version contains supplementary material available at 10.1186/s12974-021-02119-w.

## Background

Preterm infants are frequently exposed to both prenatal and postnatal inflammatory events, generally due to infectious agents. Chorioamnionitis is often the first event and is the cause of 50% of preterm births < 28 weeks gestation [1–5]. Many of these infants develop fetal inflammatory response syndrome (FIRS) [5], a severe, innate immune reaction to the mother’s infection that results in major consequences to the preterm infant in spite of the brain not being directly infected. These include higher mortality, greater neonatal morbidity, and higher rates of long-term neurocognitive disability than preterm infants without FIRS [4–11].

Preterm infants also commonly experience postnatal systemic inflammatory illnesses such as early-onset sepsis (EOS), late-onset sepsis (LOS), or necrotizing enterocolitis (NEC). The incidence of EOS ranges between 2 and 4% [3, 12–16], LOS between 16 and 31% [12, 14–17], and NEC between 5 and 10% [12–16]. Similar to infants with FIRS, long-term neurodevelopmental outcomes for infants that experience postnatal inflammatory events without direct infection of the brain are worse than for infants that do not [15, 18].

Intact neurocognitive outcomes, especially long-term learning and memory, are dependent on developmental integrity of the hippocampus, which begins rapid development at 28 weeks post-conceptional age [19, 20]. The preterm hippocampus is particularly vulnerable to injury from adverse conditions including inflammation [21–23]. Preterm infants exposed to chorioamnionitis have smaller volume hippocampi compared to preterms without chorioamnionitis exposure [23]. A similar reduction of hippocampal volume occurs in preclinical models of chorioamnionitis [24]. The functional consequence of smaller hippocampal volumes in former preterm infants is poor working memory at 2 years of age [25]. Thus, it is likely that systemic inflammation is a key contributor to poor preterm infant outcomes that are associated with hippocampal development; however, the mechanism by which inflammation affects the hippocampus is not well understood.

To address this question, we previously studied the inflammatory response of the hippocampus to a prenatal inflammatory event in a rat model of FIRS and reported persistent postnatal altered expression of pro-inflammatory genes and microglial activation associated with gray matter dysregulation [26]. In the current study, we assessed how a postnatal inflammatory challenge in the setting of FIRS would affect the hippocampus with the hypothesis that a prenatal inflammatory event would modulate the response to the postnatal event.

Immune training (sensitization) and tolerance (desensitization) [27, 28] describe conditioning such that either exaggerated or suppressed responses occur, respectively, to a second exposure [27]. This phenomenon is well established in adult models, but understudied across the prenatal-postnatal spectrum such that a prenatal exposure may affect inflammatory responses to a secondary postnatal exposure. One recent study demonstrated that prenatal exposure to an inflammatory challenge with the viral mimic poly I:C early in gestation results in decreased expression of TNFα, IL-6, and IFNγ and increased expression of IL-1β when an LPS challenge is given at adulthood [29], suggesting that immune conditioning effects are long-lived. Whether immune training or tolerance responses occur acutely in the postnatal period following late gestation prenatal inflammation, such as FIRS, has not been studied.

While the combination of prenatal and postnatal inflammation is common in preterm infants, data on outcomes in infants experiencing both events are limited. In this study, we challenged FIRS-exposed neonatal rats with postnatal LPS to better understand how prenatal inflammation affects the hippocampal tissue and glial cell immune response to a postnatal inflammatory challenge within the first week of life.

## Methods

### Animals

Animals were studied with the approval of the Institutional Animal Care and Use Committee at the University of Minnesota. Timed-pregnant Sprague-Dawley rat dams were purchased (Charles River, Wilmington, MA) and housed for 5 days before interventions and 1 week before birth of pups. Rats were housed in a temperature and humidity-controlled animal care facility with 12 h:12 h light:dark cycle and allowed food and water ad libitum.

### Model of fetal and postnatal inflammation

On gestational day 20 (term = 22 days) dams underwent laparotomy to facilitate intra-amniotic (i.a.) injections to model FIRS as previously reported [26]. Dams were premedicated with long-acting buprenorphine (1.2 mg/kg) 4 h prior to surgery. Under general anesthesia with isoflurane (3% for induction and 1.5% for maintenance) via facemask, a laparotomy was performed. Each dam was randomly assigned to receive doses of 1 μg of LPS (*Escherichia coli* O155:B5, Sigma, St. Louis, MO) dissolved in 50 μL normal saline (NS) to model FIRS or an equivalent volume of NS to each amniotic sac. After uterine horns were exposed, every amniotic sac was injected with the assigned treatment. Abdominal incisions were closed in two layers with nylon sutures, staples, and tissue glue. Incisions were treated along their lengths with bupivacaine (2 mg/kg s.c.). Delivery was performed operatively on gestational day 22 as reported [26]; dams were under general anesthesia with isoflurane. Following delivery, dams were killed using an overdose of sodium pentobarbital (100 mg/kg, i.p.). Each pup was removed from the amniotic sac and resuscitated by clamping the umbilical cord, drying with gauze, and placing on a warming pad. Mouths of pups were gently opened repeatedly to stimulate breathing until skin color became pink. Pups were then placed in the nest of a foster dam. Weights were obtained at postnatal days (P) 5 and 7. To model the effect of postnatal inflammatory episodes, pups were randomly administered either LPS i.p. (0.1 μg/kg) or an equivalent volume of saline on P5 and then returned to the nest [30, 31]. Four experimental groups were created: prenatal saline-postnatal saline (SS), prenatal LPS-postnatal saline (LS), prenatal saline-postnatal LPS (SL), and prenatal LPS-postnatal LPS (LL).

### Tissue and cell preparation

Tissue was collected from equal numbers of male and female pups at P7 after overdose of sodium pentobarbital (100 mg/kg, i.p.). For pups used for qPCR and protein analysis, the brain was removed and both hippocampi were quickly dissected on ice, flash-frozen in liquid nitrogen, and stored at – 80 °C until analysis. Pups used for immunohistochemistry underwent transcardial perfusion-fixation before removal of brain as previously described [26]. Microglia were isolated from whole brain tissue using the Gentle MACS Octo Dissociator with Heaters and Neural Tissue Dissociation Kit (Miltenyi Biotech, Auburn, CA). Whole brains were digested according to the manufacturer’s protocol for animal age ≤ P7 to obtain single cell preparations. Debris removal and red blood cell lysis steps were performed according to Adult Brain Dissociation Kit protocol (Miltenyi Biotech) following completion of Neural Tissue Dissociation protocol. Microglia were obtained by positive selection using CD11b/c beads and magnetic separating column based on manufacturer’s protocol (Miltenyi Biotech).

### Quantitative RT-PCR

qPCR experiments were performed as previously described (*n* = 6–8) [26]. Total RNA was isolated from hippocampi or isolated microglia using an RNA extraction kit (Thermo Fischer Scientific, Waltham, MA and Qiagen Inc., Germantown, MD, respectively) and cDNA was obtained using 500 ng of RNA in a high capacity RNA to cDNA kit (Applied Biosystems, Foster City, CA). The qPCR experiments were performed using 4 μL of diluted cDNA and 0.5 μL × 20 Taqman primer/probe (Applied Biosystems, Supplementary Table 1). Each sample was assayed in duplicate and normalized against ribosomal protein S18 (hippocampi) or RPP30 (isolated microglia). Reference genes were selected following analysis of 8 genes in RefFinder [32].

### Immunohistochemistry

CD11b and GFAP immunohistochemistry was performed to identify microglia and astrocytes respectively as previously described (*n* = 5–7) [26]. Sections were incubated with mouse monoclonal anti-rat CD11b/c (ab1211, 1:1000; Abcam) or rabbit polyclonal GFAP (ab7260, 1:500, Abcam) followed by anti-mouse and anti-rabbit biotinylated secondary antibody and avidin-horseradish peroxidase conjugate solution (Vector Laboratories, Burlingame, CA). The protein/antibody complex was visualized using DAB (Vector Laboratories). For co-localization experiments, hippocampal slides were incubated overnight at 4 °C with goat Iba-1 (011-27991,1:500, Wako) and mouse MHCII (Ab23880,1:50, Abcam). They were subsequently incubated at room temperature for 2 h with Alexa Fluor 488 donkey anti-goat IgG (H+L) (A11055, 1:200, Invitrogen) and Alexa Fluor 555 donkey anti-mouse IgG (A31570, 1:200, Invitrogen). The slides were cover-slipped using Vectashield PLUS (Vector Laboratories).

To determine area and activated microglial cell counts, four × 20 hippocampal images (CA1 and CA3 bilaterally) were obtained from a single section. Microglia and astrocyte areal coverage ratios in the hippocampus were measured as has been reported [26]. Using set intensity thresholds, three measurements of non-stained pixels were made of each image and then averaged. The area of CD11b or GFAP positive cells was calculated by subtracting non-stained pixels from total pixel area (Photoshop, Adobe, San Jose, CA). The mean was calculated among four images for each animal and then each treatment group mean was established. Areal coverage ratio was measured by dividing CD11b or GFAP positive area by total area of the image. The number of activated hippocampal microglia was determined by counting CD11b+ and Iba1+ cells with thickened bodies and blunted processes at equivalent sites for each animal [33]. The mean was calculated for each animal and then each treatment group mean was established. Co-localization cell counts were obtained from six hippocampal images (CA1, CA2, and CA3 bilaterally). The total number of double positive cells was determined for each animal and the mean was calculated for each treatment group. The scorer was blinded to animal ID and group assignments for area calculation and cell counting.

### Western analysis

Cytoplasmic and nuclear protein from individual frozen hippocampi were extracted using the EpiQuik Nuclear Extraction Kit (EpiGentek, Farmingdale, NY) per manufacturer’s protocol. The protein was quantified with a Bradford assay (Bioworld, St. Louis Park, MN) after which reducing agent (Thermo Scientific, Waltham, MA) and LDS (Thermo) was added to the samples to prepare the protein for gel separation. Twenty micrograms of protein per sample was separated on NuPage 4–12% Bis-Tris Gels (Thermo) and then transferred to a nitrocellulose membrane (Millipore, Burlington, MA). The membranes were blocked in Rockland (Pottstown, PA) Blocking Buffer for Fluorescent Western Blotting for 1 h at room temperature and incubated overnight at 4 ^o^C with primary antibodies. After incubation with the secondary antibodies for 45 min at room temperature, the membranes were imaged and analyzed with Odyssey infrared scanning (LiCor Bioscience, Lincoln, NE). Target proteins were standardized to β-actin or α-tubulin for cytoplasmic protein and histone deacetylase 1 (HDAC1) for nuclear protein. Primary antibodies were purchased from Cell Signaling Technologies (Danvers, MA) against: p65 (8242S, 1:1000), p50 (13586S, 1:1000), IκBα (4812S, 1:500), IκBβ (94101S, 1:1000), β-actin (5356S, 1:50,000), HDAC1 (5356S,1:50,000), and α-Tubulin (3873S, 1:50,000). Secondary antibodies: Alexa Fluor 680 conjugated anti-mouse IgG (1:12,500, Jackson Laboratory, Bar Harbor, ME), Alexa Fluor 790 conjugated anti-rabbit IgG (1:12,500, Jackson Laboratory).

### Statistical analysis

Data are reported as mean ± SEM. All comparisons were specified a priori. One-way ANOVA was used to test multigroup comparisons with Tukey’s multiple comparison tests (GraphPad Prism v6, La Jolla, CA). Statistical significance was accepted at *p* < 0.05.

## Results

### Neonatal outcomes

**Fig. 1:**
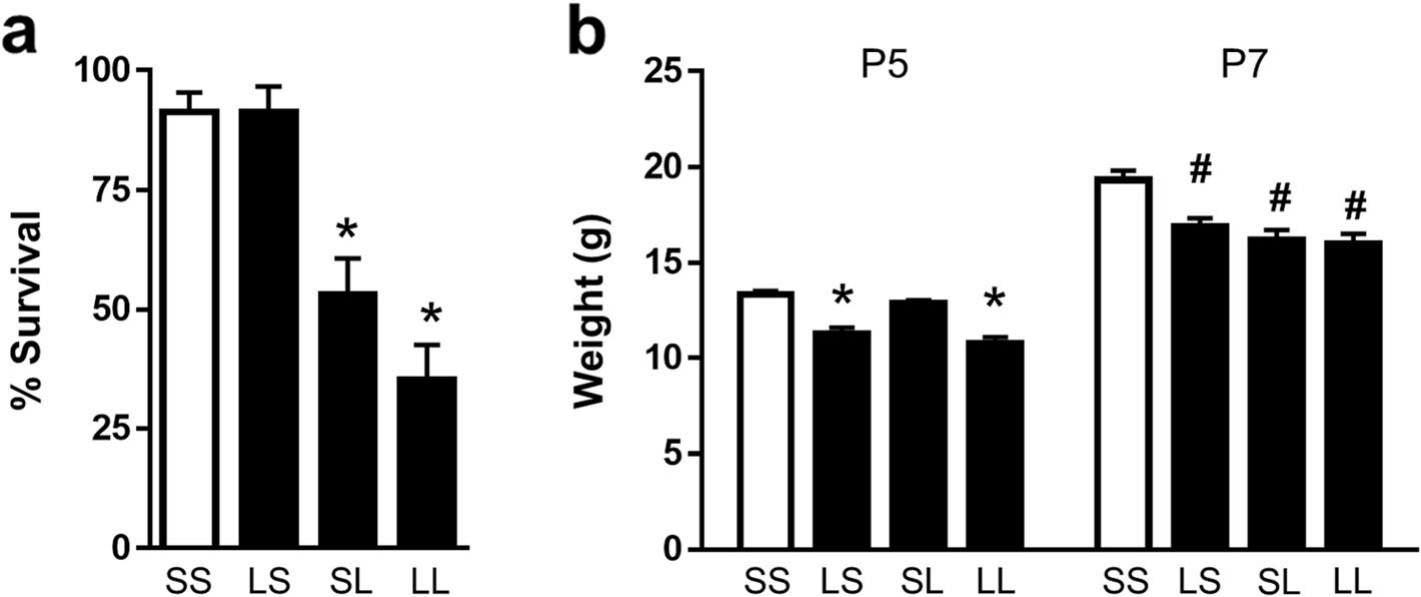
Effect of FIRS and postnatal inflammation on survival and growth. Rat pups were exposed to prenatal i.a. LPS (L) or saline (S) (listed first) and then postnatal i.p. LPS or saline at P5 (listed second) to create 4 treatment groups. **a** The percentage of pups from each litter surviving at 7 days of life following fostering is depicted (*n* = 20–22 litters). There was significantly lower survival in both groups that received postnatal LPS (SL and LL); prenatal treatment did not change survival outcomes (*p < 0.05 vs SS and LS). **b** Weights of pups that received i.a. LPS (LS and LL) were decreased on postnatal (P) day 5 prior to postnatal i.p. LPS injection (*p < 0.05 vs SS and SL; *n* = 12–30). At P7, all groups that received LPS prenatally or postnatally had lower weights than the controls (^#^*p* < 0.05 vs SS; *n* = 6–17). Data presented as mean ± SEM

Survival was measured among the four treatment groups on P7 following i.p. LPS or saline administered on P5 (Fig. 1a). We previously showed no difference in survival between neonatal animals exposed to prenatal LPS or saline [26]. Pups receiving postnatal LPS had increased mortality compared to both groups receiving postnatal saline. Prenatal treatment did not affect survival. There was minimal mortality after P7 for all groups. Growth at P5 and P7 was affected for animals that received prenatal or postnatal LPS (Fig. 1b). We previously showed that birthweights were lower for animals that received prenatal LPS [26]. Among the four treatment groups, both prenatal LPS treated groups (LS, LL) had poorer weight gain at P5 (prior to postnatal injection) than both prenatal saline groups. At P7, LPS treatment administered prenatally, postnatally or at both time points caused a decrease in weight compared to controls, but were not different among each other.

### Hippocampal inflammatory mediators

**Fig. 2:**
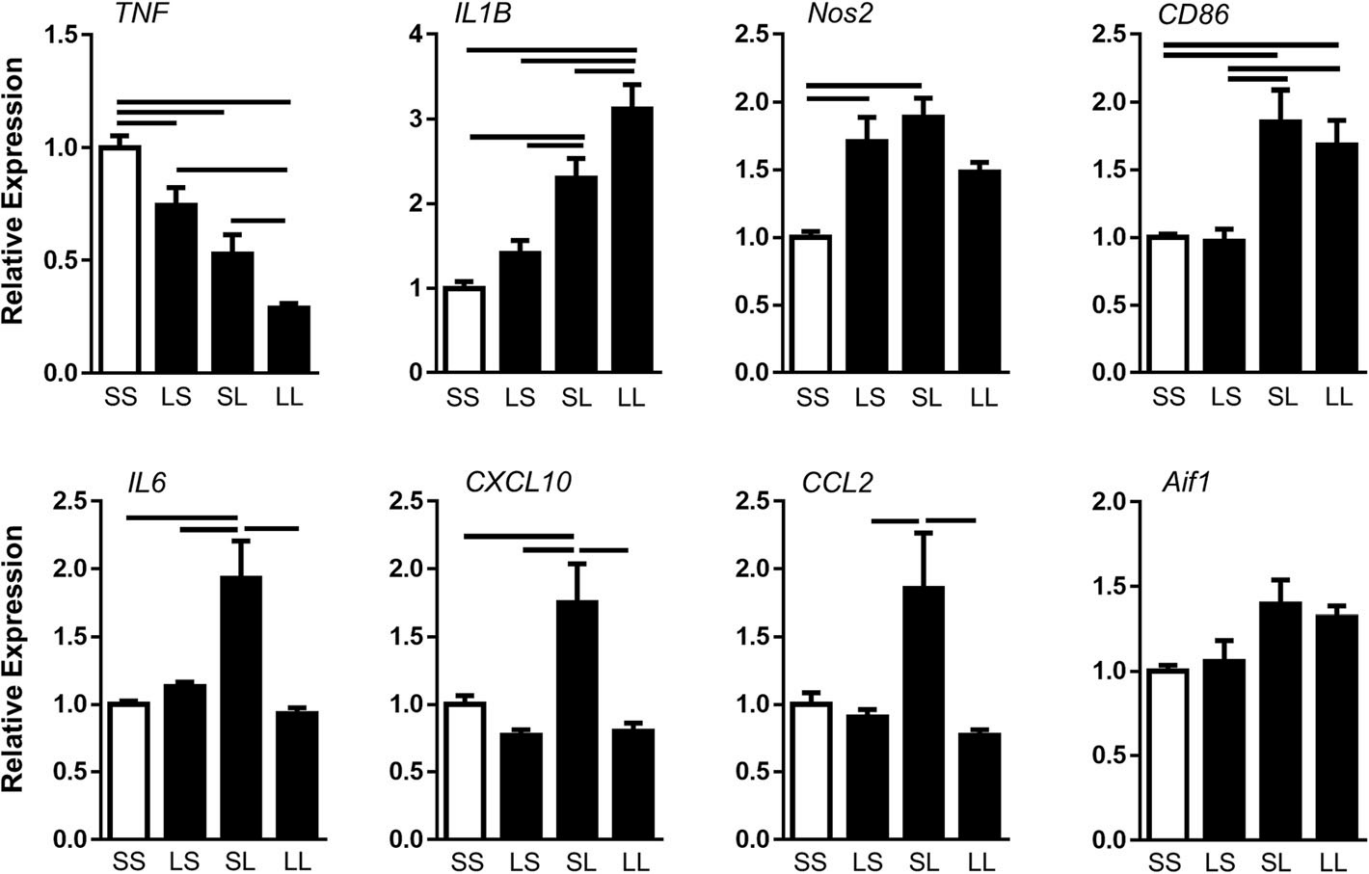
Effect of FIRS and postnatal inflammation on hippocampal gene expression of pro-inflammatory mediators at P7. Rat pups were exposed to prenatal i.a. LPS (L) or saline (S) (listed first) and then postnatal i.p. LPS or saline at P5 (listed second) to create four treatment groups. Bars above the graphs indicate significant differences between groups (*p* < 0.05). Data presented as mean ± SEM. Genes are normalized to ribosomal protein S18

To determine how pre- and postnatal LPS challenge altered the hippocampal immune response at P7, we measured transcription of several inflammatory genes (Fig. 2). Expressions of *IL1B* (gene for IL-1β), *NOS2* (gene for iNOS), *CD86*, *CXCL10*, *IL6*, and *CCL2* were all upregulated 1.5–2-fold in the SL group compared to controls. In contrast, *CXCL10*, *IL6*, and *CCL2* were all downregulated by ~ 50% in the LL group compared to the SL group. *Aif1*, the gene for Iba1, demonstrated a similar pattern, but did not reach significance. *NOS2* expression in the LL group was not significantly different from the SL group in the four-group comparison, but also not significantly different from the control group. *CD86* was not different between the two postnatal LPS groups (SL, LL). In contrast, *IL1B* was further upregulated in the LL group by 1.3-fold compared to the SL group. *TNF*, the gene for cytokine TNFα, responded differently from other inflammatory markers to pre- and postnatal LPS treatment with a 50% downregulation in the SL group compared to the control group. However, similar to several other markers, the LL group was also downregulated by 50% compared to the SL group.

Hippocampal anti-inflammatory markers are shown in Supplementary Fig. 1a. Significant hippocampal gene expression differences across groups were limited to IL10, a cytokine responsible for suppressing macrophage function [34]. *IL10* was downregulated by 50% in the LS group and 30% in the LL group compared to controls, differences that are consistent with increased pro-inflammatory mediators. There were no differences in expression of *Arg1* (the gene for arginase), *MRC1* (the gene for CD206) or *STAT6*. Overall, prenatal LPS alters the hippocampal inflammatory gene response to postnatal LPS.

### Hippocampal NFκB regulation

**Fig. 3:**
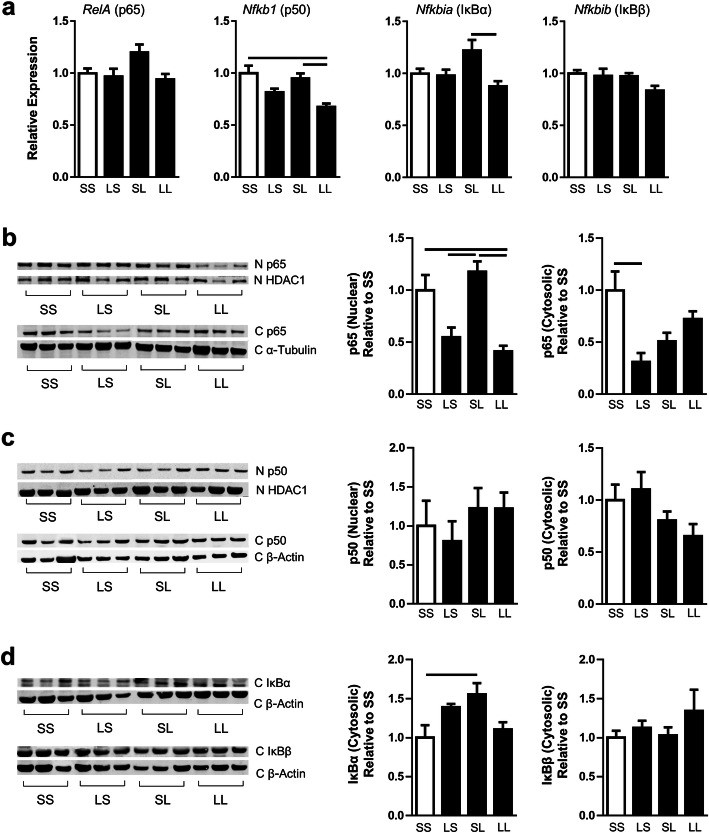
Effect of FIRS and postnatal inflammation on NFκB pathway gene expression and protein in the hippocampus at P7. Rat pups were exposed to prenatal i.a. LPS (L) or saline (S) (listed first) and then postnatal i.p. LPS or saline at P5 (listed second) to create four treatment groups. **a** Relative gene expression of indicated NFκB pathway genes in the hippocampus at P7 normalized to ribosomal protein S18 (*n* = 5–7/group). Protein product of each gene is in parentheses. **b** Western blot analyses of nuclear and cytosolic protein fractions of NFκB p65 in the hippocampus at P7 (*n* = 3–6/group). **c** Western blot analyses of nuclear and cytosolic protein fractions of NFκB p50 in the hippocampus at P7 (*n* = 4–6/group). **d** Western blot analyses of cytosolic fractions of IκBα and IκBβ proteins in the hippocampus at P7 (*n* = 3–5/group). In all graphs, bars above the graphs indicate significant differences between groups (*p* < 0.05). Data presented as mean ± SEM

We measured the effect of pre- and postnatal LPS on the NFκB pathway as a further measure of immune response in the hippocampus (Fig. 3). NFκB is a transcription factor activated by several upstream signaling pathways to regulate transcription of pro-inflammatory cytokines. The NFκB family consists of five proteins from which dimers are formed; the most abundant dimer is composed of p65 and p50 [35]. Dimers translocate from the cytoplasm to the nucleus to initiate transcription of target genes. We found that gene expression of *NFkb1*, the gene for the p50 subunit, in the LL group was downregulated by 30% compared to control and SL treatment groups (Fig. 3a). There were not significant differences of *RelA* expression, the gene for the p65 subunit, in the four-group comparison. We measured p65 and p50 protein by western blot in both the nucleus and cytosol as an indication of functional activity (Fig. 3b, c). The p65 subunit protein was decreased by 65% in the nuclear fraction of the LL group compared to the SL group (Fig. 3b). Again, attenuation of the response to postnatal LPS was demonstrated when a prenatal LPS dose was administered. The p65 nuclear findings were accompanied by a 70% decrease of cytosolic p65 in the LS group compared to controls. There was a non-significant trend decrease in the SL group compared to controls. We did not find differences of the p50 subunit protein in the nucleus or cytosol (Fig. 3c).

NFκB is prevented from translocating to the nucleus by its inhibitor IκB [36]. When phosphorylated by upstream effectors, IκB is degraded by ubiquitin, and NFκB is able to translocate [36]. As a further characterization of the NFκB pathway, we measured gene and protein expression of IκBα and IκBβ, two IκB proteins with similar properties but some functional differences (Fig. 3a, d) [37]. We found that gene expression of *Nfkbia*, the gene for IκBα, was downregulated by 30% in the LL group compared to the SL group (Fig. 3a). The protein expression of cytosolic IκBα was increased by 1.5-fold in the SL group compared to the control group (Fig. 3d). There was a non-significant trend downregulation of IκBα in the LL group compared to the SL group. There were no significant differences in IκBβ gene or protein expression among the groups.

### Microglial activation

**Fig. 4:**
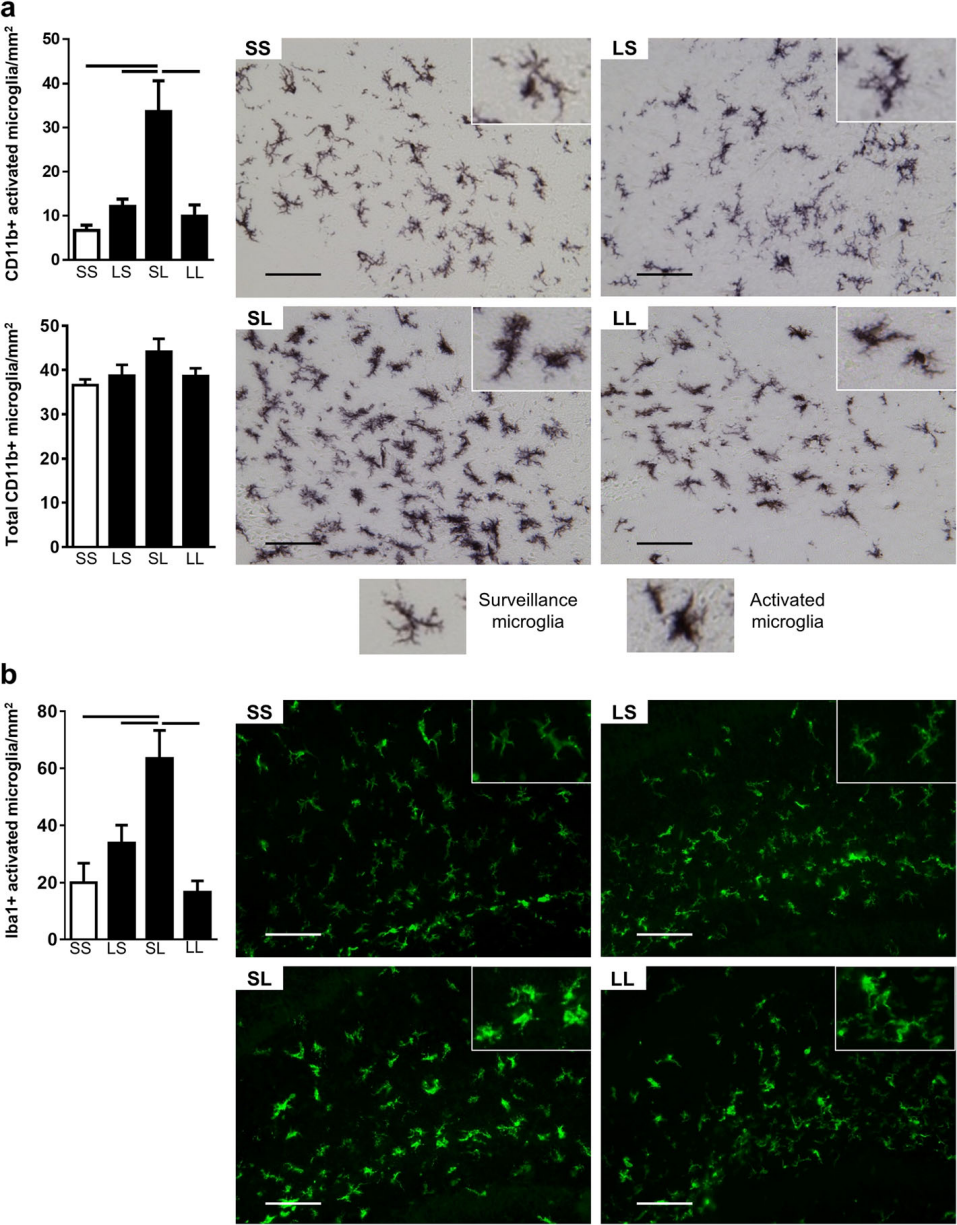
Postnatal LPS increases activated microglia in the hippocampus, but the effect is attenuated by FIRS. Rat pups were exposed to prenatal i.a. LPS (L) or saline (S) (listed first) and then postnatal i.p. LPS or saline at P5 (listed second) to create four treatment groups. On P7, microglial activation was quantified in each treatment group by counting activated microglia and total microglia. **a** Activated microglia count (top bar graph) and total microglia count (bottom bar graph) of CD11b+ cells. Activated microglia were identified by thickened cell bodies and blunted processes. Examples of surveillance (non-activated) and activated microglia are shown below pictures. **b** Activated microglia count of Iba1+ cells identified by thickened cell bodies and blunted process. Bars above the graphs indicate significant differences between groups (*p* < 0.05; *n* = 5–7/group). Data presented as mean ± SEM. Pictures representative of each group are shown at × 20. Scale bar represents 100 μm

Microglia are the tissue-specific macrophages of the brain. They respond to pathogen-associated molecular patterns (PAMPs) through TLR4 and IFNγ receptors in a characteristic cell response known as microglial activation to produce cytokines, present antigen, and induce adaptive immunity [38, 39]. Histologically, activation is measured by increased density and altered morphology [26, 33, 40]. We measured microglial activation in the hippocampus, as a response to pre- and postnatal inflammation, using CD11b and Iba1 immunohistochemistry at P7 (Fig. 4). The number of CD11b+ (Fig. 4a) and Iba1+ (Fig. 4b) cells with a thickened cell body and blunted processes were counted. There was a 5- and 3-fold increase in the number of activated microglia measured by CD11b+ or Iba1+ cells, respectively, in the SL group compared to the SS group. In contrast, the LL group had 70% fewer activated microglia (CD11b+ and Iba1+) than the SL group. The total number of CD11b+ cells did not differ among groups. To account for complex morphology, we also calculated the cross-sectional areal coverage of CD11b+ cells as a ratio of total image area (Supplementary Fig. 2). Areal measurement yielded similar findings as the cell counts. There was a 1.4-fold increase in microglial area in the SL group compared to the SS group. Prenatal LPS prior to postnatal LPS prevented this activation. Prenatal LPS appears to prevent the microglial activation response that follows postnatal LPS alone.

### Astrocyte activation

**Fig. 5:**
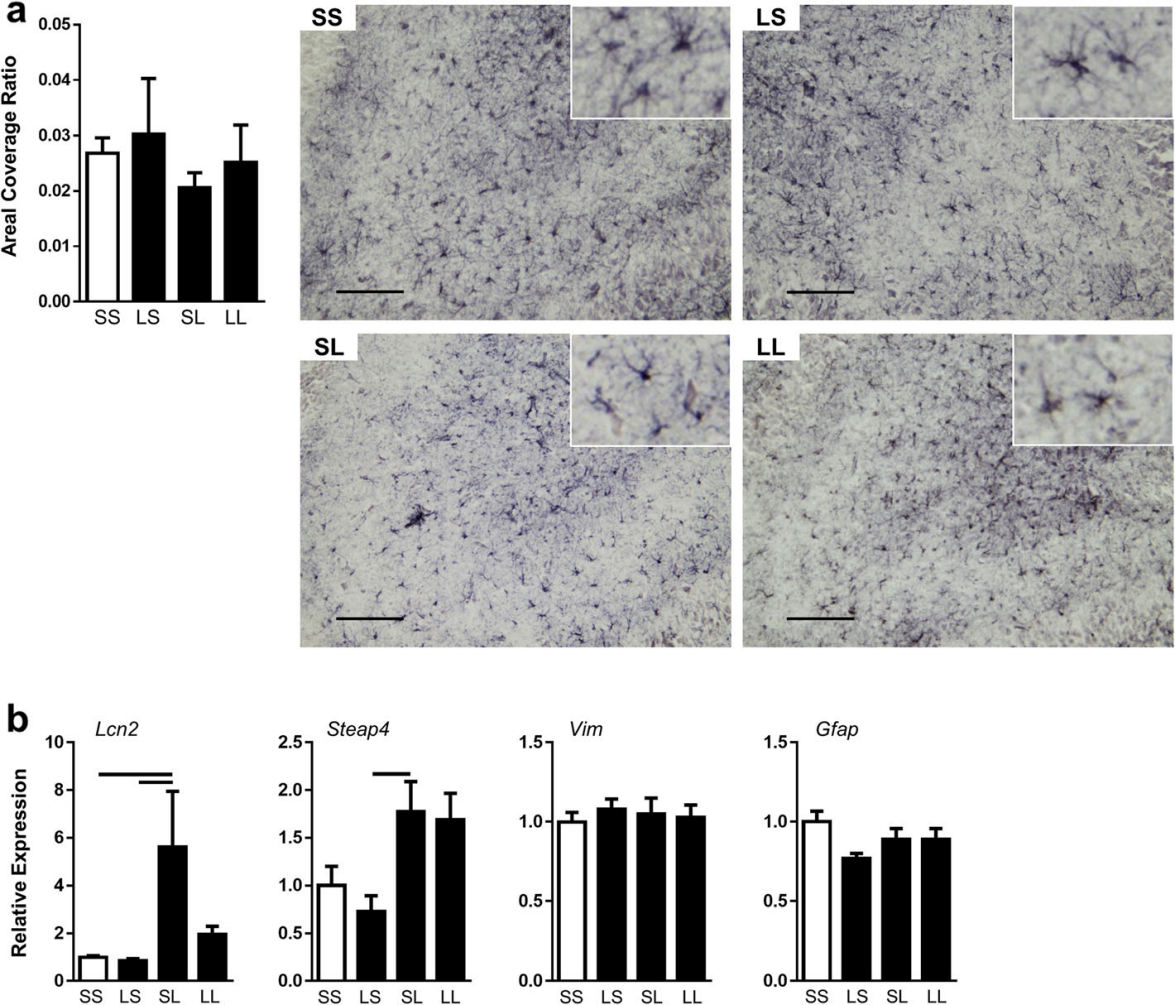
Effect of FIRS and postnatal inflammation on astrocytes in the hippocampus at P7. Rat pups were exposed to prenatal i.a. LPS (L) or saline (S) (listed first) and then postnatal i.p. LPS or saline at P5 (listed second) to create four treatment groups. **a** On P7, reactive astrocytosis was quantified in each treatment group by calculating areal coverage ratio of Gfap+ cells in the hippocampus. Scale bar represent 100 μm. Pictures representative of each group are shown at × 20. **b** Expression of reactive astrocyte genes in the hippocampus at P7. Bars above the graphs indicate significant differences between groups (*p* < 0.05). Data presented as mean ± SEM

We measured cross-sectional area coverage ratio of Gfap+ astrocytes in the hippocampus at P7 to determine changes in astrocyte reactivity following pre- and postnatal inflammatory challenge (Fig. 5a). We found no differences among treatment groups in amount of Gfap. We also measured the expression of multiple genes associated with astrocyte activation (Fig. 5b) [41]. *Lcn2* expression was increased by 5.5 fold in the SL group compared to the control group. There was a trend decrease in the LL group compared to the SL group in a pattern similar to our measurements of microglial activation. *Steap4* expression demonstrated a trend increase in the SL group compared to controls. There were no expression differences in *Vim* or *Gfap*.

### Microglia-specific inflammation

**Fig. 6:**
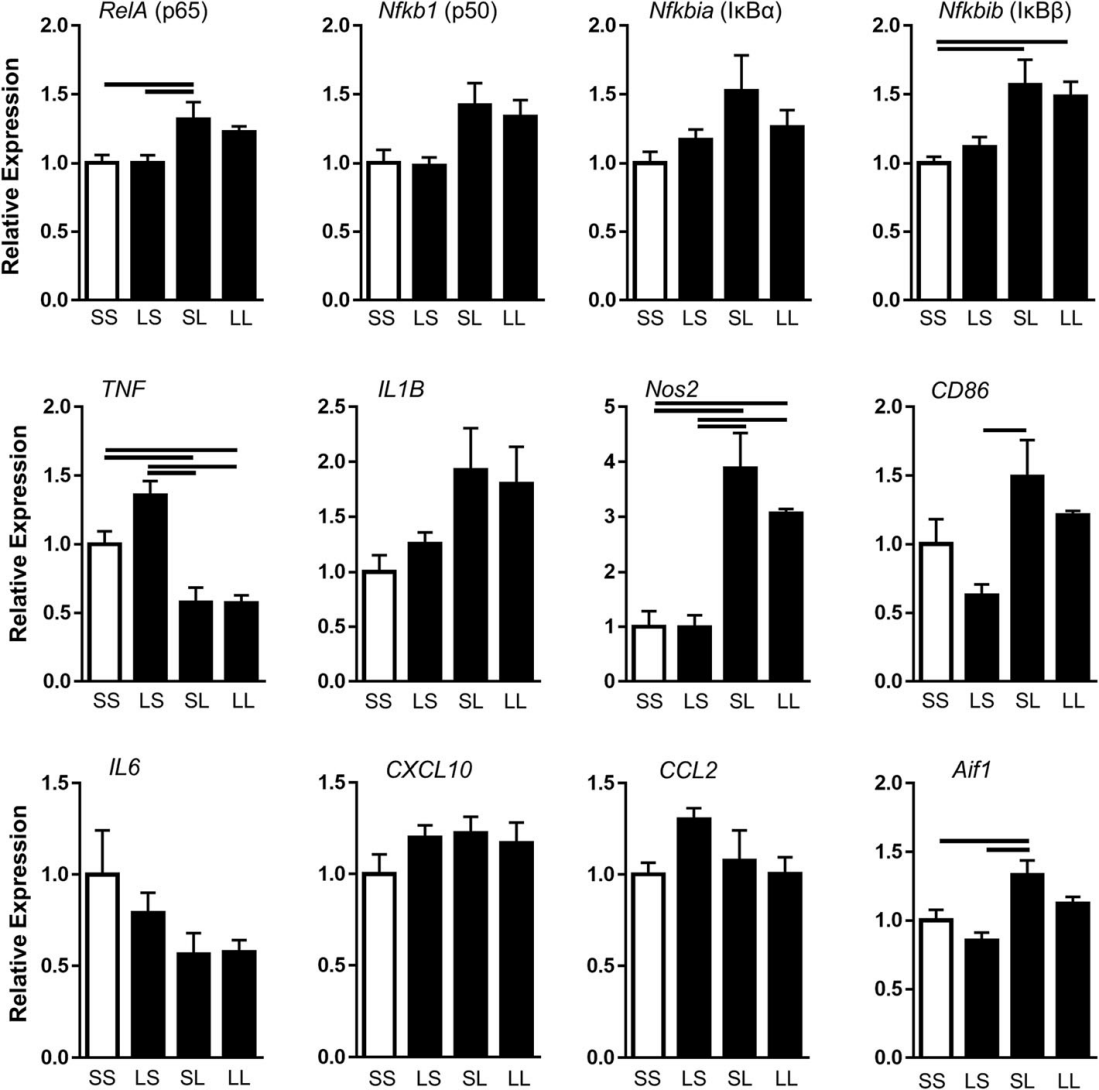
Effect of FIRS and postnatal inflammation on gene expression of M1 activation markers in isolated microglia at P7. Rat pups were exposed to prenatal i.a. LPS (L) or saline (S) (listed first) and then postnatal i.p. LPS or saline at P5 (listed second) to create four treatment groups. Bars above the graphs indicate significant differences between groups (*p* < 0.05). Data presented as mean ± SEM. Genes are normalized to the ribonuclease RPP30

Microglial activation in response to pathogens is commonly described as M1 activation and is characterized by increased transcription factor activation (e.g., NFκB and STAT1), increased expression of surface markers (e.g., CD86, MHCII), increased iNOS to produce NO for pathogen killing, and increased production of cytokines (e.g., IL-1β, TNFα, IL-6) and chemokines (e.g., CCL2 and CXCL10) [38, 42]. Based on hippocampal expression patterns of these genes and morphologic changes signifying microglial activation, we sought to determine whether microglia expressed similar immune tolerance and training gene expression and thus the major contributor to these responses in the hippocampus (Fig. 6). In the NFκB pathway, there was a 1.3-fold upregulation of *RelA* and 1.5-fold upregulation of *Nfkbib* in the SL group compared to controls. *Nfkb1* demonstrated a similar trend upregulation in the SL group. *Nos2*, *CD86*, and *Aif1* were upregulated in the SL group compared to SS and LS groups. There was a trend increase of *IL1B* in the SL group compared to the SS group. *TNF* was downregulated by postnatal LPS in both SL and LL groups. Differential expression was not measured between SL and LL groups, indicating prenatal LPS did not affect response to postnatal LPS of these genes in microglia. Other pro-inflammatory cytokines and chemokines did not demonstrate significant differences.

M2 activation is the microglial response to repair tissue and dampen inflammation and is characterized by anti-inflammatory markers such as IL-10, Arg1, and CD206 [38, 39]. Among the anti-inflammatory markers measured in isolated microglia, only *IL10* displayed a difference (Supplementary Fig. 1b). The SL group was downregulated in comparison to the LS group, but not different compared to SS or LL groups.

**Fig. 7:**
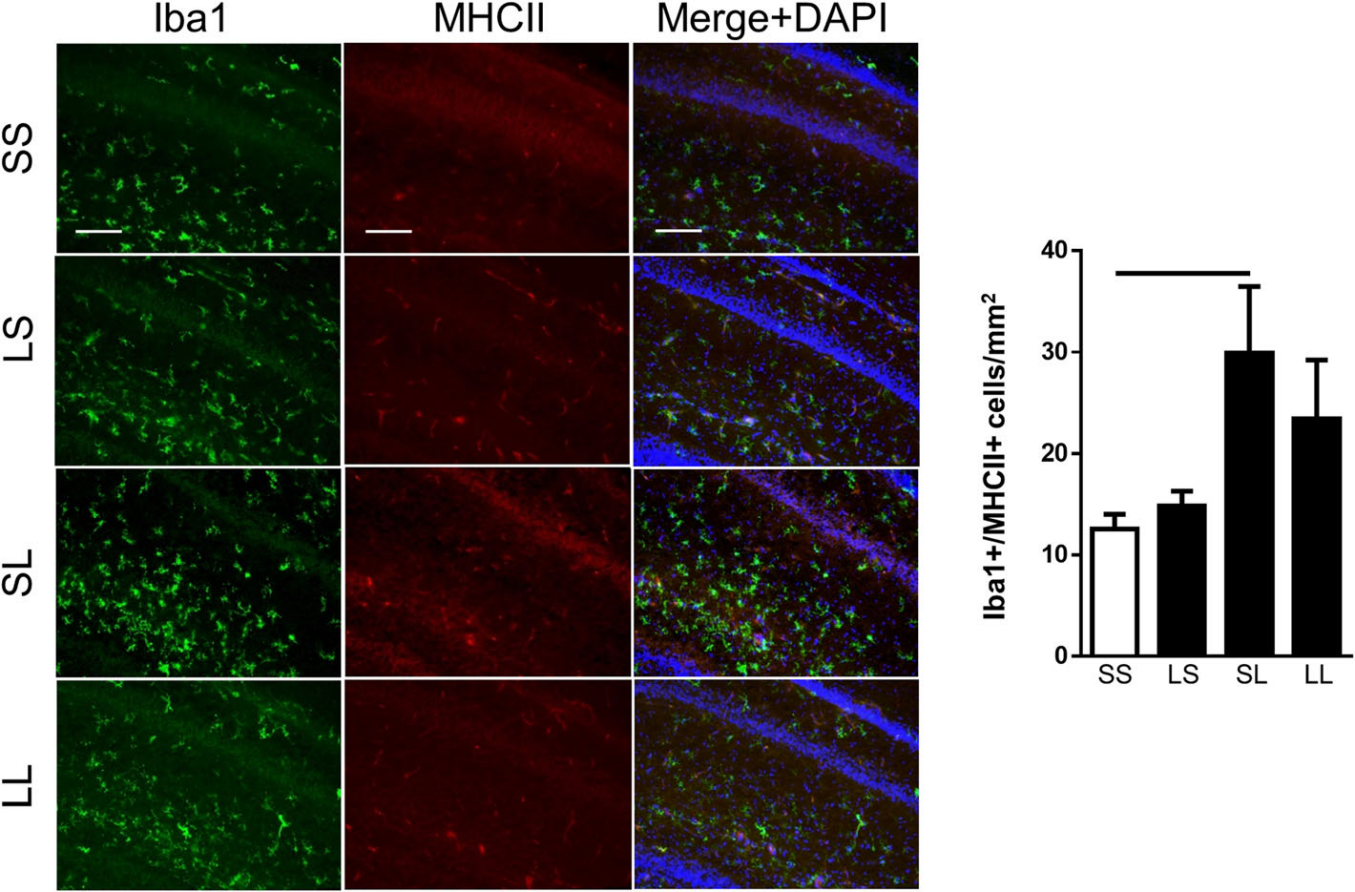
Co-localization of M1 marker MHCII with Iba1+ microglia in the hippocampus at P7. Rat pups were exposed to prenatal i.a. LPS (L) or saline (S) (listed first) and then postnatal i.p. LPS or saline at P5 (listed second) to create four treatment groups. Total double positive Iba1 (green) and MHCII (red) cells were quantified in each treatment group (blue = DAPI). Bars above the graph indicate significant differences between groups (*p* < 0.05; *n* = 4–6/group). Data presented as mean ± SEM. Pictures representative of each group are shown at × 20. Scale bar represents 100 μm

Co-localization of MHCII+ cells with Iba1+ microglia was quantified to further measure activated microglia in the hippocampus and correlate with upregulation of M1 activation markers (Fig. 7). Iba1+MHCII+ cells increased by 2.5 fold in the SL group compared to the control group indicating activation caused by postnatal LPS. Prenatal LPS did not cause differences between the SL and LL groups although LL was also not different from the controls.

## Discussion

FIRS and postnatal inflammation are independently linked to long-term neurodevelopmental deficits for preterm infants [11, 15]. We sought to better understand how inflammatory exposures occurring both pre- and postnatally affect immune tolerance and training in the hippocampus. The hippocampus was chosen because of its role in learning and memory, its rapid development in the late fetal/early neonatal time period, and the prominence of learning and memory deficits in preterm infants. We found that pro-inflammatory mediator gene expression, microglial activation, and astrocyte activation gene expression increased following postnatal inflammation. We also found signals of hippocampal immune tolerance; there was an attenuation of many of these effects when prenatal inflammatory challenge was administered prior to postnatal challenge. Although histologic microglial activation caused by postnatal LPS was attenuated by prenatal LPS, gene expression in isolated microglia did not demonstrate a similar immune tolerance.

Whether repeated exposure to inflammatory events causes immune training or tolerance appears to be dependent on timing. Adult mice receiving repeated dosing of i.p. LPS over four consecutive days have decreasing concentrations of plasma and brain cytokines after each dose, suggesting immune tolerance [28]. LPS tolerance is long-lived in the brain after systemic injection to adults and neonatal mice [43, 44]. LPS dosing to adult mice repeated after 270 days results in attenuated inflammatory response, including IL-1β and IL-6 in the hippocampus [43]. Similarly, systemic LPS to 10-day-old mice results in downregulated expression of *IL1B* and *TNF* in the hypothalamus after adult LPS challenge [44]. In contrast, prenatal LPS followed by LPS injection at adult ages resulted in exaggerated (immune training) expression of *IL1B*, *TNF*, and *IL6* in the hippocampus [45]. Our study is unique in that we studied how prenatal LPS dosing affects hippocampal response to postnatal LPS in the acute neonatal period. Despite prenatal exposure, the results are consistent with other studies in showing that LPS induces immune tolerance in the brain.

Mechanisms that underlie the attenuated hippocampal responses caused by prenatal inflammation are not known. Because NFκB has a central role in inflammation, we explored the possibility that p50 could be involved because the p50/p50 homodimer is a repressor of inflammatory response [46]. The p50 subunit has no transcriptional activity itself and is typically a regulator of activity when paired with other subunits [47]. In contrast, the p65/p50 heterodimer is a transcription factor that promotes pro-inflammatory responses [35]. In our model, decreased nuclear p65 protein following pre- and postnatal LPS without a parallel change in p50 is consistent with an inhibitory phenotype and resulted in the decreased expression of several downstream inflammatory markers. Changes in hippocampal inflammation appear to be dependent on p65 rather than p50 and responsible mechanisms likely occur upstream from IκB and NFκB phosphorylation.

Whether hippocampal immune tolerance is beneficial or harmful to neonates is not known. Neonatal inflammation is a well-established mediator of brain injury resulting in poorer behavioral outcomes in humans and preclinical models [6–10, 24, 48, 49]. Preclinical studies show that attenuation of the neuroinflammatory response, by inducing tolerance or inhibiting inflammatory cell activity, is associated with improved developmental outcomes [43, 50–52]. In contrast, harmful effects may result from multiple inflammatory exposures. For example, exaggerated IL-1β response following multiple doses of LPS is associated with adverse learning and memory behavioral consequences [53, 54]. In our model, expression of *IL1B* demonstrated an immune training response that was consistent with these studies despite other signals of hippocampal immune tolerance. Regulation of IL-1β in the hippocampus is important for proper development of learning and memory [53]. Absent or excessive IL-1β results in impairments [55, 56]. Alterations of IL-1β, such as that seen in our model, may lead to long-term learning impairment.

Microglial activation is also associated with poor neurodevelopmental outcomes [50, 51]. In rodent models, microglial activation is associated with long-term learning impairments, motor deficits, and anxiety, results that mirror several domains of poor neurodevelopmental outcomes of infants born preterm [57–59]. Under normal developmental conditions, microglia have critical homeostatic functions that include synaptogenesis, axonal elongation, promotion of myelination, growth factor production, and they are the primary source for IL-1β during normal hippocampal-dependent learning [45, 53, 60, 61]. These functions are likely disrupted by microglial activation.

We hypothesized that immune tolerance signals in hippocampal tissue gene and protein expression were a reflection of microglia based on CD11b and Iba1 histologic findings and therefore isolated microglia to explore that possibility. Gene expression in microglia in the SL group was similar to expression in the hippocampus for several M1 activation markers suggesting that microglia are an important driver of the acute hippocampal inflammatory response. There was similar upregulation of *Nos2*, *CD86*, *IL1B*, and *Aif1*, and similar downregulation of *TNF*. We also measured upregulation of *RelA* and *Nfkbib* in the NFκB pathway. However, there were two notable differences between hippocampal tissue and microglia suggesting that microglia are not the only cells contributing to inflammatory response. First, unlike in the hippocampus, we did not measure significant attenuation of expression in any marker in microglia when prenatal challenge was administered prior to a postnatal challenge. Second, whereas *IL6*, *CXCL10*, and *CCL2* demonstrated robust upregulation in the SL group and attenuation in the LL group in the hippocampus, these genes were unaffected in microglia by pre- or postnatal LPS.

The upregulation of M1 markers in SL microglia is consistent with the significant increase in histologic-activated microglia that we measured by CD11b and Iba1. In contrast, there was significant attenuation of histologic activated microglia in the LL group, but without a similar attenuation of M1 marker expression in microglia. This suggests different phenotypes of microglia between SL and LL groups. Single cell gene studies have shown numerous phenotypes of microglia [62]. Several factors could influence this difference in phenotype. We considered that upregulation of M2 marker expression in microglia in the LL group could inhibit morphologic changes caused by postnatal inflammation. However, there were no significant differences between SL and LL groups in the M2 genes that we measured. Another possibility is that exogenous signaling of IL-6, CXCL10, and CCL2 influence microglial activation in this model. They are highly upregulated in the SL hippocampus, attenuated in the LL hippocampus, both in a pattern similar to CD11b and Iba1 results. Neurons are one possible exogenous source. Preclinical models show that neurons under adverse conditions produce CCL2 [63, 64] and CXCL10 [65, 66] that increase microglial activation. IL-6 is also produced by neurons but less studied [67].

Astrocytes are another potential source of *IL6*, *CXCL10*, and *CCL2* expression. Similar to microglia, astrocytes undergo a transformation to reactive astrocytosis in response to injury and disease. Reactive astrocytes have a gene expression pattern distinct from non-reactive astrocytes [41], produce pro-inflammatory cytokines by an NFκB-mediated mechanism [52, 68, 69], and have immune tolerance responses [68, 70]. To determine whether astrocytes may contribute to hippocampal inflammatory mediator results and demonstrate immune tolerance in our pre- and postnatal LPS model, we measured Gfap+ astrocytes in the hippocampus. In contrast to microglia, we found no histological differences in Gfap+ astrocytes among treatment groups. However, upregulation of *Lcn2* and *Steap4,* in addition to *CXCL10,* genes associated with reactive astrocytosis [41], suggest a change in astrocyte reactivity following postnatal inflammatory challenge. *Lcn2* and *CXCL10* also demonstrated immune tolerance in the LL group.

Reactive astrocyte gene expression is likely a direct effect of activated microglia. Liddelow et al. demonstrated that activated microglia are required to induce transformation of cultured A1 reactive astrocytes (nomenclature similar to M1 microglia) from non-reactive astrocytes after 7 days [41]. Our study is consistent with this process because (1) outcomes measured 2 days after postnatal LPS administration was a timeframe adequate to measure a robust histologic microglial activation but only a partial astrocyte gene response; (2) the responses of Lcn2 and CXCL10 to pre- and postnatal LPS mirror hippocampal responses.

Non-resident immune cells including infiltrating macrophages and neutrophils could also contribute to the hippocampal immune response in this model, but their impact is likely much less than microglia and astrocytes. A study by Hutton et al. used a preterm fetal ovine model to identify infiltrating macrophages by morphology 72 h following inflammatory challenge [71]. They identified 60–100 macrophages per mm^2^ in white matter areas. In contrast, they identified 1–3 macrophages per mm^2^ in the hippocampus. We did not specifically identify infiltrating cells in our study, but found a far greater density of activated microglia in the hippocampus that in the similar model. Infiltrating cells may be a more significant mediator of inflammation in white matter regions than gray matter regions such as the hippocampus.

## Conclusions

Postnatal LPS administered to neonatal rats at P5 to model sepsis resulted in a pro-inflammatory immune response in the hippocampus. Evidence of immune tolerance resulted if prenatal LPS to model FIRS was administered first. Microglia demonstrate a robust inflammatory response to postnatal LPS, but partial immune tolerance response. Hippocampal immune tolerance likely results due to the responses of multiple cell types.

## Supplementary Information

**Additional file 1: Supplementary Figure 1**. Effect of FIRS and postnatal inflammation on gene expression of anti-inflammatory mediators in the hippocampus and isolated microglial at P7. Rat pups were exposed to prenatal i.a. LPS (L) or saline (S) (listed first) and then postnatal i.p. LPS or saline at P5 (listed second) to create four treatment groups. (a) Relative expression measured in hippocampus. (b) Relative expression measured in isolated microglia. Bars above the graphs indicate significant differences between groups (p<0.05). Data presented as mean ± SEM. Genes are normalized to S18 (hippocampus) or RPP30 (microglia).

**Additional file 2: Supplementary Figure 2**. Postnatal LPS increases activated microglia in the hippocampus, but the effect is attenuated by FIRS. Rat pups were exposed to prenatal i.a. LPS (L) or saline (S) (listed first) and then postnatal i.p. LPS or saline at P5 (listed second) to create four treatment groups. On P7, microglial activation was quantified in each treatment group by calculating areal coverage ratio of CD11b+ cells in the hippocampus. Bars above the graph indicate significant differences between groups (p<0.05). Data presented as mean ± SEM.

**Additional file 3: Supplementary Table 1**. List of Taqman Probes.

## Data Availability

All data generated or analyzed during this study are included in the published article or supplementary files.

## Abbreviations

Aif1: Allograft inflammatory factor 1
Arg: Arginase
BDNF: Brain-derived neurotrophic factor
CCL2: C-C motif chemokine ligand 2
CXCL10: C-X-C motif chemokine ligand 10
EOS: Early-onset sepsis
FIRS: Fetal inflammatory response syndrome
Gfap: Glial fibrillary acidic protein
HDAC1: Histone deacetylase 1
iNOS: Inducible nitric oxide synthase
i.a.: Intra-amniotic
i.p.: Intraperitoneal
Lcn2: Lipocalin 2
LL: Prenatal LPS-postnatal LPS group
LOS: Late-onset sepsis
LS: Prenatal LPS-postnatal saline group
NEC: Necrotizing enterocolitis
NS: Normal saline
PAMP: Pathogen-associated molecular patterns
P(x): Postnatal day x
SL: Prenatal saline-postnatal LPS group
Steap4: Steap 4 metalloreductase
SS: Prenatal saline-postnatal saline group
Vim: Vimentin
