# Supplementary figures and images for: Fetal inflammation induces acute immune tolerance in the neonatal rat hippocampus
Source: J Neuroinflammation. 2021 Mar 11;18:69. doi: 10.1186/s12974-021-02119-w (PMC7953777; doi:10.1186/s12974-021-02119-w)

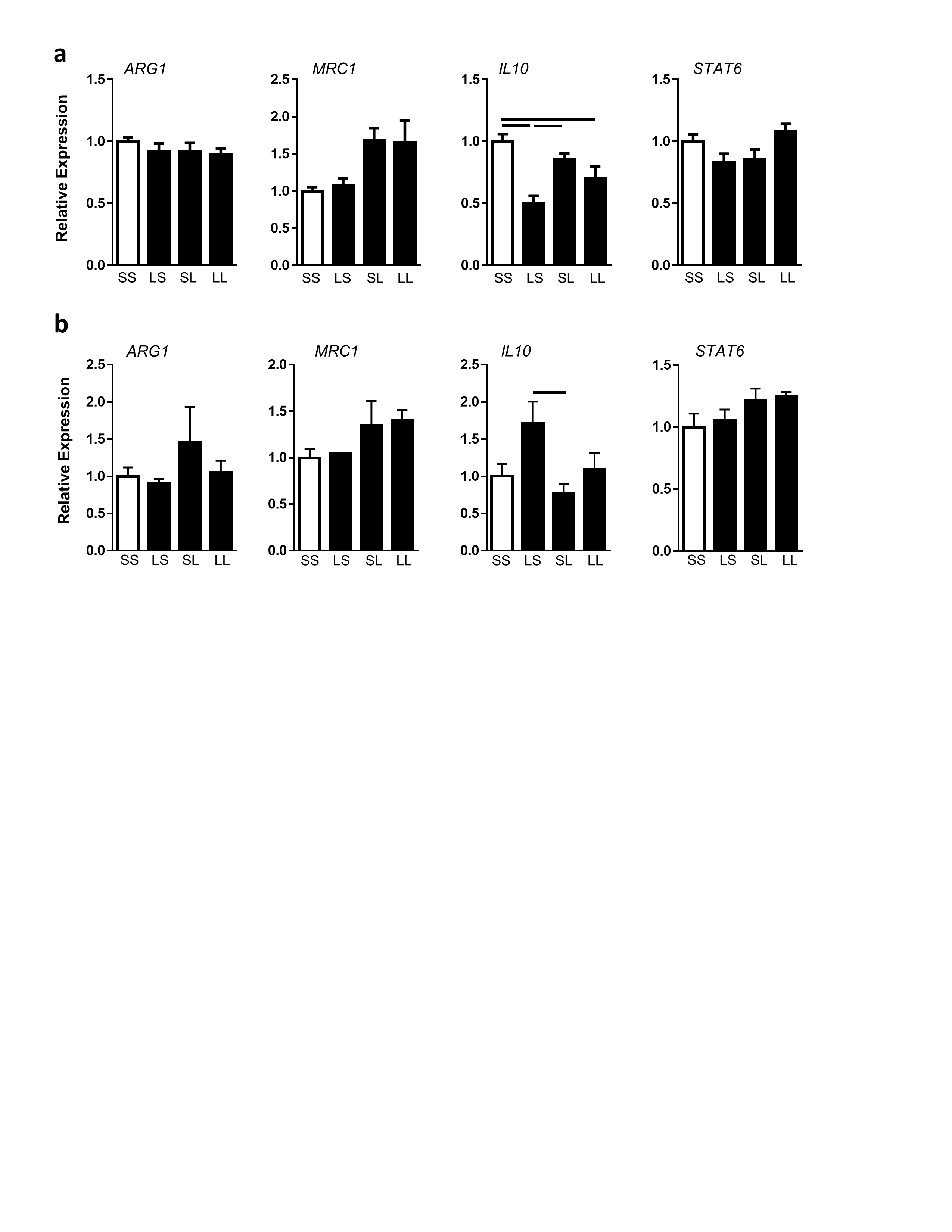

Supplement: Supplementary file 1 — Additional file 1: Supplementary Figure 1. Effect of FIRS and postnatal inflammation on gene expression of anti-inflammatory mediators in the hippocampus and isolated microglial at P7. Rat pups were exposed to prenatal i.a. LPS (L) or saline (S) (listed first) and then postnatal i.p. LPS or saline at P5 (listed second) to create four treatment groups. (a) Relative expression measured in hippocampus. (b) Relative expression measured in isolated microglia. Bars above the graphs indicate significant differences between groups (p<0.05). Data presented as mean ± SEM. Genes are normalized to S18 (hippocampus) or RPP30 (microglia). [file 12974_2021_2119_MOESM1_ESM.tif]

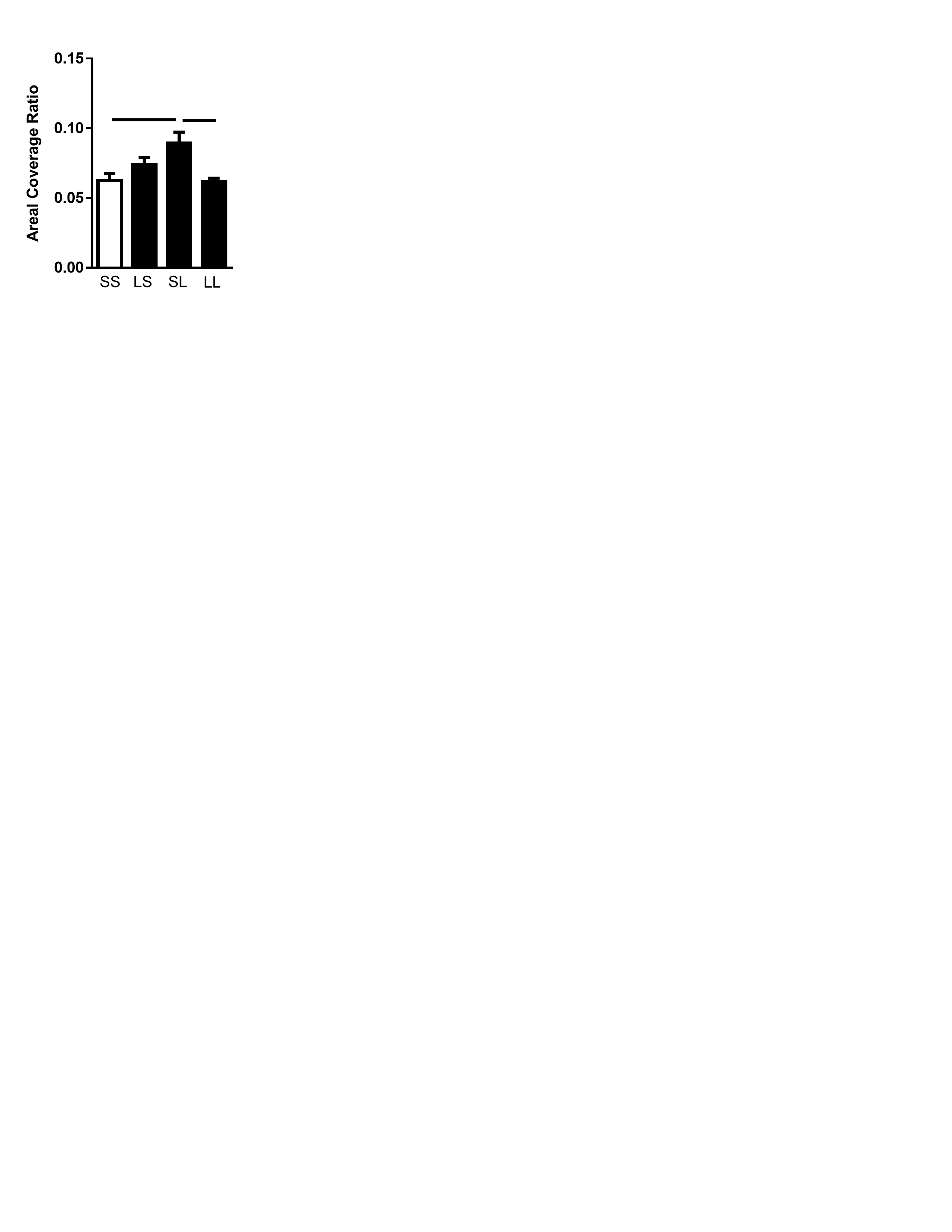

Supplement: Supplementary file 2 — Additional file 2: Supplementary Figure 2. Postnatal LPS increases activated microglia in the hippocampus, but the effect is attenuated by FIRS. Rat pups were exposed to prenatal i.a. LPS (L) or saline (S) (listed first) and then postnatal i.p. LPS or saline at P5 (listed second) to create four treatment groups. On P7, microglial activation was quantified in each treatment group by calculating areal coverage ratio of CD11b+ cells in the hippocampus. Bars above the graph indicate significant differences between groups (p<0.05). Data presented as mean ± SEM. [file 12974_2021_2119_MOESM2_ESM.tif]
