# Supplementary material for: Fetal inflammation induces acute immune tolerance in the neonatal rat hippocampus
Source: J Neuroinflammation. 2021 Mar 11;18:69. doi: 10.1186/s12974-021-02119-w (PMC7953777; doi:10.1186/s12974-021-02119-w)
Supplement: Supplementary file 3 — Additional file 3: Supplementary Table 1. List of Taqman Probes. [file 12974_2021_2119_MOESM3_ESM.docx]

| **ID** | **Gene ID** | **Taqman Assay ID** |
| --- | --- | --- |
| Arginase | Arg1 | Rn00691090_m1 |
| CCL2 | CCL2 | [Rn00580555_m1](https://www.thermofisher.com/taqman-gene-expression/product/Rn00580555_m1?CID=&ICID=&subtype=) |
| CD206 | MRC1 | Rn01487342_m1 |
| CD86 | CD86 | [Rn00571654_m1](https://www.thermofisher.com/taqman-gene-expression/product/Rn00571654_m1?CID=&ICID=&subtype=) |
| CXCL10 | CXCL10 | Rn00594648_m1 |
| Gfap | Gfap | Rn01253033_m1 |
| Iba1 | Aif1 | Rn005745125_g1 |
| IκBα | Nfkbia | [Rn01473657_g1](https://www.thermofisher.com/taqman-gene-expression/product/Rn01473657_g1?CID=&ICID=&subtype=) |
| IκBβ | Nfkbib | [Rn00578384_m1](https://www.thermofisher.com/taqman-gene-expression/product/Rn00578384_m1?CID=&ICID=&subtype=) |
| IL-1β | IL1B | [Rn00580432_m1](https://www.thermofisher.com/taqman-gene-expression/product/Rn00580432_m1?CID=&ICID=&subtype=) |
| IL- 6 | IL6 | [Rn01410330_m1](https://www.thermofisher.com/taqman-gene-expression/product/Rn01410330_m1?CID=&ICID=&subtype=) |
| iNos | NOS2 | [Rn00561646_m1](https://www.thermofisher.com/taqman-gene-expression/product/Rn00561646_m1?CID=&ICID=&subtype=) |
| Lcn2 | Lcn2 | Rn00590612_m1 |
| p50 | Nfkb1 | [Rn01399572_m1](https://www.thermofisher.com/taqman-gene-expression/product/Rn01399572_m1?CID=&ICID=&subtype=) |
| p65 | RelA | [Rn01502266_m1](https://www.thermofisher.com/taqman-gene-expression/product/Rn01502266_m1?CID=&ICID=&subtype=) |
| RPP30 | RPP30 | Rn01479850_m1 |
| S18 | S18 | Rn01428915_g1 |
| STAT6 | STAT6 | Rn01505881_m1 |
| Tnfα | Tnf | [Rn99999017_m1](https://www.thermofisher.com/taqman-gene-expression/product/Rn99999017_m1?CID=&ICID=&subtype=) |
| Steap4 | Steap4 | Rn01755355_m1 |
| Vim | Vim | Rn00667825_m1 |

Supplementary Table 1: List of Taqman Probes
